# Handheld dynamometer reliability to measure knee extension strength in rehabilitation patients—A cross-sectional study

**DOI:** 10.1371/journal.pone.0268254

**Published:** 2022-05-17

**Authors:** João Pinto-Ramos, Tiago Moreira, Frederico Costa, Helena Tavares, João Cabral, Cristina Costa-Santos, Joana Barroso, Bernardo Sousa-Pinto

**Affiliations:** 1 Department of Physical Medicine and Rehabilitation, Centro Hospitalar Universitário São João, Porto, Portugal; 2 CINTESIS – Center for Health Technologies and Services Research, University of Porto, Porto, Portugal; 3 MEDCIDS – Department of Community Medicine, Information and Health Decision Sciences, Faculty of Medicine, University of Porto, Porto, Portugal; 4 Department of Biomedicine, Faculty of Medicine, University of Porto, Porto, Portugal; 5 i3s – Institute for Health Research and Innovation, University of Porto, Porto, Portugal; 6 Departments of Neuroscience and Physical Medicine and Rehabilitation, Northwestern University, Feinberg School of Medicine, Chicago, Illinois, United States of America; Universiti Sains Malaysia, MALAYSIA

## Abstract

**Introduction:**

The Handheld Dynamometer (HHD) has the potential to overcome some of the logistic and economic limitations of isokinetic dynamometers for measuring knee extension muscle strength. However, its reliability has not been fully assessed. The purpose of this study is to measure intra and inter-rater reliability of HHD for knee extension strength in patients receiving rehabilitation treatment, as well as to understand in which conditions is the reliability higher.

**Methods:**

Twenty-nine patients admitted in an inpatient Physical Medicine and Rehabilitation unit were consecutively included in this cross-sectional study. Two experienced and two inexperienced physicians made two assessments of knee extension strength with HHD, separated by three hours. Intraclass Correlation Coefficients (ICC), absolute differences between assessments, and correlations between strength and functional variables were calculated.

**Results:**

Intra and inter-rater ICC were overall high (≥ 0.950 and 0.927, respectively). Higher values were found when average of two measurements were made for estimating intra-rater ICC (ICC = 0.978; 95%CI = 0.969–0.985) but not for inter-rater ICC. ICC were not statistically significantly different when calculated based on measurements performed by inexperienced physicians and experienced ones. There was a moderate correlation between strength and functional variables.

**Conclusion:**

Handheld Dynamometer seems to be a reliable option to measure knee extension muscle strength, particularly when two measurements are performed and their average is reported.

## Introduction

Functional disability is a major problem in global population, affecting mobility and independent living. According to the Centers for Disease Control and Prevention, one-quarter of all adults in the United States have some kind of disability; fourteen percent have severe difficulty walking and climbing stairs, and seven percent have difficulty doing daily tasks without assistance [1]. In Portugal, forty percent of population reported some kind of long-term disability [2].

Functional disability is difficult to measure, one of the reasons being its parameters are dependent on patients’ experiences and expectations. Moreover, patient reported outcome measures (such as the 36-Item Short-Form Survey) [3] and functional tests (such as the 6 minutes walking test) [4] are time-consuming and its application challenging in most outpatient clinical rehabilitation settings due to lack of an easy clinical measurable outcome that can be obtained along clinical treatment. This prompts the need for methods simultaneously capable to (i) accurately and reliably assess patients’ disability (and its evolution over time), and (ii) be easily implemented in the clinical practice, namely in the outpatient setting of rehabilitation programs. Such methods may include the measurement of muscle strength, since a negative correlation between muscle strength, functional disability and activity of daily living dependence has been shown in the past [5–7]. In this context, measuring knee extension strength may be particularly adequate, as it displays a key role for maintenance of functional capacity for activities as walking, sitting, dressing or having a shower [8–10].

The isokinetic dynamometer (ID) is the gold-standard method for evaluating muscle strength in knee extension, but it is difficult to apply in the clinical practice, since it is very expensive, non-portable and requires previous specialized training [11]. On the other hand, the handheld dynamometer (HDD) is a less expensive and portable device that can also be used to measure muscle strength; its use requires less training, potentially rendering it more applicable in clinical practice [11]. However, questions still remain on the accuracy of HDD. In fact, while some studies have shown a high correlation between measurements in ID and HDD [11–13], only few of them calculated sample size or used more than 2 observers [12, 14–19].

The purpose of this work is to measure the intra-observer and inter-observer reliability of HHD for the assessment of knee extension strength. In addition, this study aims to analyse if the reliability of HHD differs according to the experience of observers, the number of measurements, and if there is an association between knee extension strength measured by HHD and functional capacity.

## Methods

### Study design

**Fig 1:**
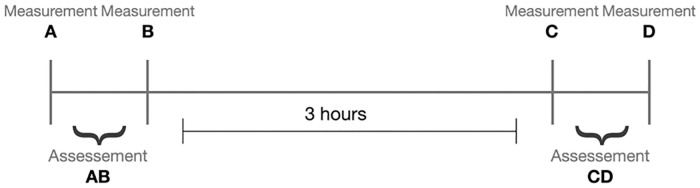
Schematic representation of the measurements performed by each observer in each participant.

Twenty-nine patients were consecutively selected for this cross-sectional study at the Inpatient unit, Physical Medicine and Rehabilitation Department, Centro Hospitalar e Universitário de São João, which is a tertiary care hospital in Northern Portugal. For each patient, four different observers, two previously experienced and two inexperienced in HHD use, made four measurements of knee extension peak force with an HHD. Do to schedule logistics and in order to systematize the measurements, two measurements were taken in early afternoon (measurement A and measurement B–assessment AB), and two three hours after (measurement C and measurement D–assessment CD) (Fig 1). All observers were physicians with at least two years of clinical experience. Intra-rater and inter-rater intraclass Correlation Coefficients (ICC), absolute differences between measurements within each assessment, and correlation with functional variables were calculated. The study was approved by Ethical Committee of the respective hospital (Research Project 289/20). Patients were asked for written consent. This article was written according to STROBE statement guidelines [20].

### Setting and participants

We assessed participants which had already been included in a previous study from our research group, with sample size of thirty patients being calculated. (Pinto-Ramos J et al., Submitted) In brief we consecutively included all patients admitted at our Service from March to June 2021 which satisfied defined inclusion and exclusion criteria. Patients aged ≥ 18 years-old performing rehabilitation treatment for any cause fulfilled the inclusion criteria. Exclusion criteria were: patients unable to ambulate without assistance before hospitalization, morbid obesity, actual lower limb bone fractures, serious pressure or venous ulcers, cardiorespiratory instability, major psychiatric disorders, severe cognitive impairment or neurologic conditions such as multiple sclerosis, traumatic brain injury or severe stroke. Patients admitted to our acute rehabilitation service were hospitalized due to acute events which have impacted their functional capacity, having been transferred for personalized and intense rehabilitation programs. Each day, patients had a 24-hour rehabilitation nursery care with activities of daily living training sessions, two physical therapy sessions of one and half hours each, one occupational therapy session and, in case of need, psychology and speech therapy.

### Variables and measurements

**Fig 2:**
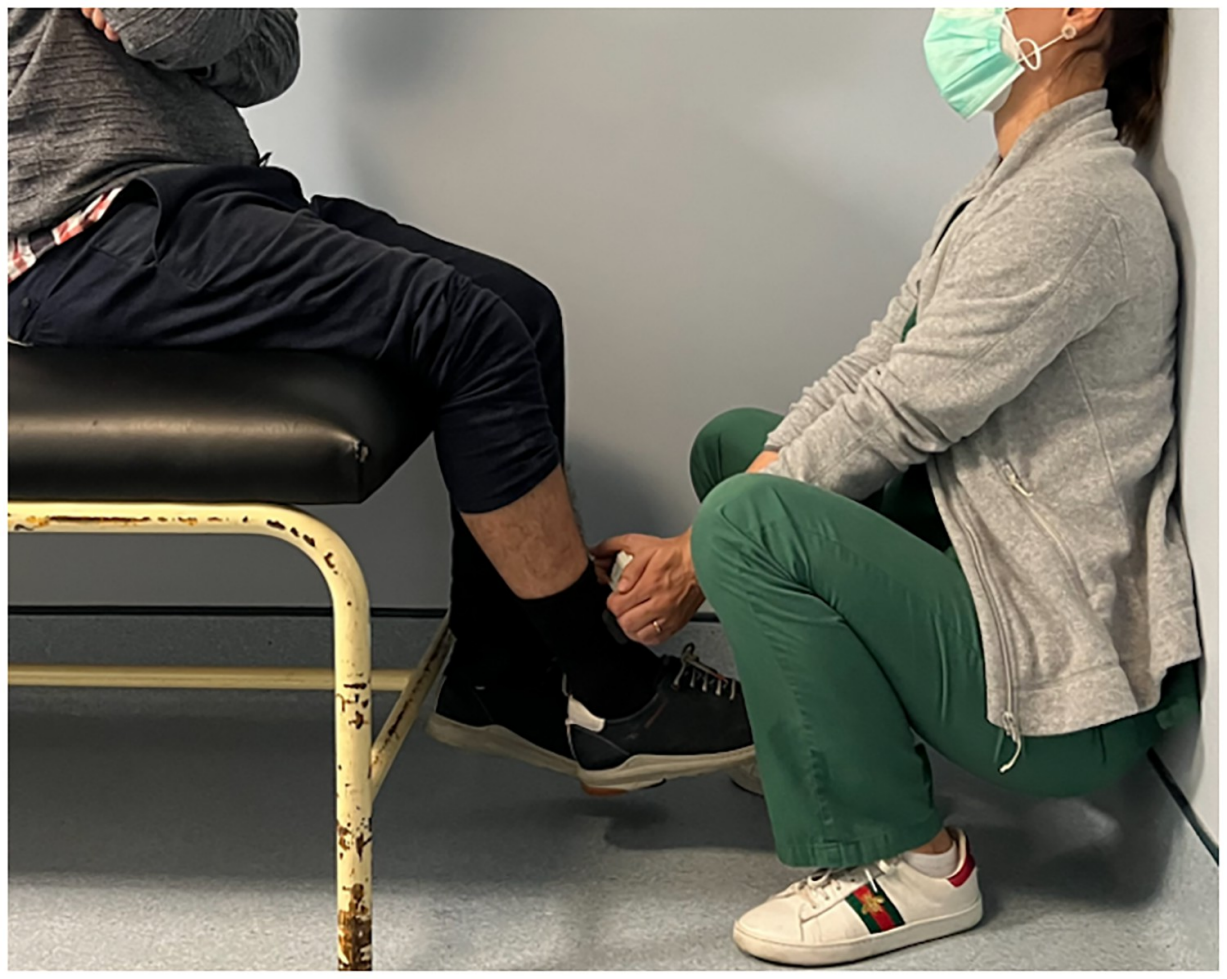
Standardized evaluation method of the patients. Observer was squatting with back against the wall and stretch arms in order to increase stability.

From each patient, knee extension strength was measured four times by each of the four observers with the HDD. That is, the four observers performed two knee extension peak force measurements with HHD (Micro FET^®^2 HHD; Hoggan Health Industries, Draper, UT, USA) at each patient (measurements A and B, comprising the assessment AB) followed by two other measurements three hours later (measurements C and D, comprising the assessment CD) (Fig 1). The three-hour interval was defined to allow some interval between assessments in order to reduce the risk of memory bias while allowing all assessments to be done in the same day (ensuring clinical stability of the patients during the interval between assessments). Patients were seated in the edge of the stretcher with the feet and hands suspended and knee flexed at 60º. The observer was in squatting position with the back against the wall for support and both arms extended to the patient dominant leg for stabilization, so that the results dependence on patient or observer strength would be minimized. The HHD was placed in the anterior leg of each patient, five centimetres above the distal part of the medial malleolus (Fig 2). Patients were asked to make and maintain maximum knee extension strength for five seconds. One-minute rest was used between measurements to decrease fatigue impact. Results displayed on HHD were registered by an independent observer, so that the patients and observers applying the HHD were blinded. Strength was measured in Newtons (N).

In order to standardize the procedures, every observer was given a thirty-minute training before the beginning of the study on how to perform HHD measurements correctly, even though two of the observers had more than two years of experience using the HHD.

From each patient, information was also collected (by a different researcher) on his/her sex and age as well as height, weight, body mass index, diagnosis, pain on visual analogue scale and current and previous modified Rankin Scale. Functional variables were also collected, namely handgrip strength, Timed Up and Go (TUG) test, 1-minute sit-to-stand (STS) test and Medical Research Council Sum Score [21–24].

### Study size

The sample size for this study was calculated simultaneously with that for the previous study. (Pinto-Ramos J et al., Submitted) Our primary effect size measure was the intra and inter-rater ICC. For us to estimate the sample size, we conducted a comprehensive literature search in order to identify previous ICC estimates. We identified seven studies providing such estimates [12, 14–19], which we pooled by random-effects meta-analysis, resulting in a pooled ICC of 0.944 (95% confidence interval (95%CI) = [0.902;0.986]) for intra-rater reliability and 0.977 (95%CI = [0.959;0.995]) for inter-rater reliability. If more than one ICC were calculated in the same study (i.e., in left and right limbs), the lower ICC value was used in the meta-analysis. The minimum sample size estimated based on those meta-analytic values using a 95%CI and a semi-width of 5% was of 13 for intra-rater and 4 for inter-rater reliability. Since the required sample size calculated for the previous study was higher (30 patients), we ended up enrolling a larger number of patients.

### Statistical analyses

For numerical (continuous) variables, we used means and standard-deviations (SD) for describing variables with normal distribution and medians and 25^th^ and 75^th^ percentiles (P25;P75) for variables with non-normal distribution. Categorical variables were described using absolute and relative frequencies (in percentage). Reliability of HHD measurements was estimated using ICC. For each set of two measurements (i.e., assessment AB, and assessment CD), we registered the average, maximum and first value. Two-way mixed-effects model ICCs were used to calculate intra-rater and inter-rater reliability. ICCs were calculated using single measurements per assessment set for maximum and first values. For intra-rater ICC, we calculated it taking in to account the measurements of all observers, comparing average, maximum and first values of the AB assessments with those of CD assessments. Individual intra-rater ICC were also presented for average, maximum and first values. For inter-rater ICC, we compared average, maximum and first values of AB assessments between observers (in an ancillary analysis, such comparisons were also performed for CD assessments). Experienced and inexperienced observers were also separately analysed for ICC comparison between different levels of training. Reliability was considered poor if ICC<0.50, moderate if 0.50≤ICC<0.75, good if 0.75≤ICC≤0.90 and excellent if ICC>0.90 [25].

Since ICC values are dependent on the heterogeneity of the population [25], absolute differences between average, maximum and first measurements of each assessment were calculated, with differences being tested using the Friedman test. We also calculated relative differences, dividing the aforementioned absolute differences by average knee extension peak force of each participant. The correlation between absolute differences and average knee extension peak force was also calculated to assess if absolute differences between assessments depend on average knee extension peak force. If correlations were found to be sufficiently strong (*r*>0.4 or *r*<-0.4), univariable linear regression models were applied.

We also estimated the correlation between average knee extension peak force of the different participants with other functional variables. Such functional tests/variables include the handgrip strength, TUG test, STS test, Medical Research Council Sum Score, actual and previous Modified Rankin, and POCUS measured rectus and quadriceps femoris muscle thickness. If correlations were found to be sufficiently strong (*r*>0.4 or *r*<-0.4), univariable linear regression models were applied, with knee extension peak force being the independent variable and the dependent variables corresponding to the results of each functional variable.

Correlations were estimated using Spearman correlation coefficients. A p-value inferior to 0.05 was considered statistically significant. The Bonferroni correction was applied in order to control for multiple comparisons. ICC values were calculated using R software and the remaining statistical analyses were done in IBM SPSS Statistics 28 (IBM, Armonk, NY).

## Results

Twenty-nine patients were included in this study. Participants’ age ranged between 19 and 82 years old with mean age of 58.8 (SD = 14.1) years old; 75.9% of patients were male (Table 1). Participants displayed a median body mass index of 23.1 kg/m^2^ (P25;P75 = 21.5;27.4), and the current modified Rankin median score was of 3 (P25;P75 = 2;4). Nineteen patients (65.5%) were not able to stand up from a chair whereas thirteen patients could not ambulate (44.8%). Patients able to ambulate had a mean TUG test of 18.9 seconds (SD = 11.6) and patients able to stand from a sitting position had a mean STS test of 17.2 (SD = 7.6). Median muscle strength was of 189.8 N (P25;P75 = 130.7;274.4).

**Table 1:** Demographic characteristics of the sample and descriptions of functional tests.

Variables	Patients (N = 29)
Age (years)–mean (SD)	58.8 (14.1)
Males–*n* (%)	22 (75.9)
BMI (Kg/m^2^)–median (P25;P75)	23.1 (21.5;27.4)
Previous mRankin–median (P25;P75)	0 (0;1)
Current mRankin–median (P25;P75)	3 (2;4)
Medical Research Council Sum Score—median (P25;P75)	46 (43;47.5)
Handgrip Strength (kg)–median (P25;P75)	18.3 (11.7;23.3)
Timed Up and Go (s)–mean (SD)^1^	18.9 (11.6)
Sit to Stand test–mean (SD)^2^	17.2 (7.6)

BMI–Body Mass Index, P25;P75–25^th^ and 75^th^ Percentile, Kg–Kilogram, m–Meter, s–Seconds, SD–Standard Deviation;

^1^–Only 16 participants could complete the test,

^2^–Only 10 participants could complete the test

The most common admission diagnoses were Intensive Care Unit Acquired Weakness (ten patients) and non-severe (Initial National Institute of Health Stroke Scale (NIHSS) <15) Stroke (six patients).

Observers rapidly adapted to the use of HHD and measurements took less than one minute in the majority of times, although positioning the most disabled patients in the stretcher was often a challenge. Also, patients with higher knee extension strength were difficult to resist in order to measure their full strength.

### Intra-rater reliability

The intra-rater ICC for knee extension strength of the 464 measurements (corresponding to 4 measurements done by each of the 4 observers to the 29 patients) was excellent with results higher than 0.950 when considering either average, maximum and first measurements. The intra-rater ICC was significantly higher when calculated based on the average of two measurements (average of AB versus average of CD) (ICC = 0.978, 95%CI = 0.969–0.985) than when based on the first measurement alone of each set (A and C) (ICC = 0.950, 95%CI = 0.928–0.965) (Table 2).

**Table 2:** Intra-rater and inter-rater intra-class correlation coefficients (ICC) of knee extension strength measured with hand-held dynamometers using average, maximum and first values within AB and CD assessments.

	ICC calculated based on average of measurements within each assessment (95%CI)	ICC calculated based on maximum of measurements within each assessment (95%CI)	ICC calculated based on first measurements within each assessment (95%CI)
Knee Extension Strength intra-rater ICC	0.978 (0.969–0.985)	0.961 (0.945–0.973)	0.950 (0.928–0.965)
Knee Extension Strength inter-rater ICC			
Assessment AB	0.932 (0.864–0.967)	0.936 (0.874–0.969)	0.927 (0.859–0.964)
Assessment CD	0.952 (0.908–0.976)	0.952 (0.911–0.976)	0.943 (0.899–0.971)

95%CI–95% Confidence Interval

Individual intra-rater ICC were similar or higher than 0.948 for the four observers, either using mean, maximum or first values (S1 Table).

### Inter-rater reliability

Considering measurements of the assessment AB, we observed an inter-rater ICC between 0.927 and 0.936 of knee extension strength measured with HHD. There were no substantial differences between ICC calculated based on average, maximum and first values of the assessment AB. (Table 2). Similar results were found from assessment CD with ICC between 0.943 and 0.952. (Table 2).

### Absolute and relative differences

The median of the absolute differences between knee extension strength assessments (AB vs CD) ranged between 15.0 and 15.4 N (depending on whether average, maximum or first measurements in each assessment were being considered). No significant differences were found between median differences calculated based on average, maximum or first measurements within each assessment (p = 0.23). Relative differences between assessments ranged between 8.7% and 10.5%. (Table 3).

**Table 3:** Absolute difference (in Newton) of knee extension strength and percentual difference of absolute difference over average muscle strength of all assessments with Hand Held Dynamometer (HHD) using mean, maximum and first measurements between assessments AB and CD for the same observer.

	Average of measurements within each assessment—Median (P25;P75)	Maximum of measurements within each assessment—Median (P25;P75)	First measurement within each assessment—Median (P25;P75)
Absolute Difference (N) Between Knee Extension Strength	15.2 (7.9;34.7)	15.0 (6.2;33.0)	15.4 (8.9;38.0)
Percentual Difference Between Absolute Difference and Average Knee Extension Strength	8.7% (5.1;16.8)	8.8% (4.5;17.4)	10.5% (4.9;18.3)

HHD–Hand Held Dynamometer, P25;P75–25th and 75th Percentiles, N–Newton;

Average HHD strength were moderately correlated with absolute differences for average (*r* = 0.477 (95%CI = 0.318;0.610) [p<0.001]), maximum (*r* = 0.415 (95%CI = 0.246;0.559) [p<0.001]) and first measurements (*r* = 0.414 (95%CI = 0.245;0.558) [p<0.001]). Univariable linear regression coefficients were subsequently applied, with coefficients being of 0.112 (95%CI = 0.080;0.144) [p<0.001] for average measurements, 0.103 (CI95% = 0.070;0.136) [p<0.001] for maximum measurements, and 0.117 (95%CI = 0.080;0.155) [p<0.001] for first measurements. On the other hand, correlations between relative differences and average HHD strength were not significant, with Spearman correlation coefficients ranging between -0.063 and -0.109.

### Experienced and inexperienced observers

Similar results were observed when comparing the reliability of measurements of experienced versus inexperienced observers. For experienced observers intra-rater ICCs were of 0.981, 0.963, 0.949, depending on whether such ICCs were estimated based on average, maximum or first measurements, respectively. For inexperienced observers, such values were of 0.976, 0.961, 0.950, respectively.

### Muscle strength and functional outcomes

Except for the Previous Modified Rankin, muscle strength measured by knee extension peak force in the HHD was moderately correlated (correlation coefficient ≥0.4) with all functional variables, including the current modified Rankin, the TUG test, the STS test, the Medical Research Council Sum Score, the Handgrip Strength and the Quadriceps and Rectus Femoris Muscle Thickness. (Table 4) Correlations after Bonferroni correction were statistically significant for all functional tests, except the Previous Modified Rankin, the Medical Research Council Sum Score and the TUG. Linear regression coefficients ranged from -0.062 (association between knee extension peak force and TUG) to 0.045 (association between knee extension peak force and STS). (Table 4).

**Table 4:** Correlation and linear regression coefficient between Handheld Dynamometer and different functional variables.

Functional Variables	Correlation coefficient (95%CI) [p-value]	Linear regression coefficient (95%CI) [p value]
Previous Modified Rankin	-0.378 (-0.660; -0.002) [0.043]*	**
Current Modified Rankin	-0.565 (-0.776; -0.239) [0.001]	-0,005 (-0,008; -0.002) [0.004]
TUG test	-0.612 (-0.854; -0.151) [0.012]*	-0,062 (-0.106; -0.019) [0.009]
STS test	0.499 (0.151; 0.737) [0.006]	0.045 (0.018; 0.073) [0.002]
Medical Research Council Sum Score	0.484 (0.132; 0.728) [0.008]*	0.019 (-0.005; 0.043) [0.109]
Handgrip Strength	0.545 (0.213; 0.765) [0.002]	0.030 (0.004; 0.055) [0.023]
Quadriceps Femoris Muscle Thickness	0.511 (0.167; 0.744) [0.005]	0.002 (0.000; 0.003) [0.012]
Rectus Femoris Muscle Thickness	0.536 (0.201; 0.759) [0.003]	0.002 (0.000; 0.005) [0.027]

95%CI–95% Confidence Interval, TUG–Timed Up and Go; STS–1-minute sit-to-stand;

*non-significant after Bonferroni correction;

** Not applicable since r<0.40

## Discussion

Our study shows that the HHD may be a reliable tool for estimating knee extensors muscle strength in rehabilitation patients, with both intra and inter-rater ICC being higher than 0.9 across all observers. Using an average of two measurements increases reliability when compared with estimates based on a single measurement, which suggests a random error in the measurements. The learning curve of HHD use is short, with experienced and inexperienced observers showing similar results.

This study has some limitations worth noting, including the fact that this is a single center study, and caution is required when generalising its results to other populations. Also, as the patients were hospitalized to perform a rehabilitation program in an acute tertiary care Hospital, results may not apply to other type of patients. Patients were observed in two assessments by four observers, so that at each set of measurements the patient was subject to eight maximum knee strength evaluations performed within a short period (being subject to sixteen of such evaluations within a period of three hours). As a result, patient fatigue may have modified the results between measurements and lead to underestimation of results. Authors tried to manage this limitation by setting a minimum one-minute rest between each measurement, so that maximum peak force could be reached every time. An additional limitation may concern the possibility of memory biases (given the three-hour gap between the two sets of measurements). This may particularly concern the possibility of psychomotor memory bias from patients. On the other hand, we believe that memory bias from the observers may not have had a relevant impact, as each day there were seven to nine patients to be assessed by each independent observer (resulting in at least 56 measurements to remember before assessment CD). Finally, since ICC values are contingent on the homogeneity of the population, results might be overestimated as a result of the heterogeneity of studied population [25]. To overcome this limitation, and in order to estimate the actual differences, absolute and percentual differences between assessments were calculated.

This work has also important strengths. Firstly, this study was designed using more than two observers, differentiating it from most other studies assessing the reliability of the HHD. Also, this study compared different ways of calculating the strength of the patient knee extension, namely using two measurements or a single measurement, in order to estimate the most reliable assessment of muscle strength. Having four observers also allowed us to include observers with different experience levels, making us able to assess whether experience associated with different results. Finally, we ensured that this study was adequately powered to obtain precise estimates.

The HHD was found to be a reliable way of measuring muscle strength, with results showing excellent reliability, in accordance with what has been previously reported in previous studies assessing other types of patients [12, 14–19].

Absolute differences between assessments were relatively low, despite being higher for patients with higher muscle strength. By contrast, the percentual differences did not vary with increase of muscle strength. This might occur because knee extension of stronger patients is possibly more difficult to resist, leading to higher errors, specially between observers with less capability to resist knee extension strength. In fact, other studies already suggested that stronger observers tend to report higher values on HHD and also that external stabilization of the HHD, namely with a belt, can be more reliable than human-resisted evaluations [19, 26–28]. Nevertheless, belt stabilization is more difficult and time-consuming, possibly rendering it impractical to apply in clinical practice.

Results were similar when considering measurements performed by both experienced and previously inexperienced observers, which suggest that the use of HHD to measure muscle strength could be generalized across physicians if a short course of training is previously done. Since this study was conducted throughout a period of four months, we cannot estimate if reliability would be this high for longer periods if observers were not trained again for standardized evaluations. Other studies with inexperienced subjects with a short course training already showed that good reliabilities could be achieved with HDD but in different muscular groups [29, 30].

Knee extension strength on HHD was moderately correlated with the majority of analysed functional variables, confirming that muscle strength, including for knee extension, may be important for these tasks, as it was also suggested by other studies [31–34]. In these participants, correlations between functional tests and muscle strength were higher than those observed between functional tests and muscle thickness (except for hand grip strength). (Pinto-Ramos J et al., Submitted) This suggests that, compared with muscle thickness, muscle strength correlates better with functional variables for this specific population, which can be justified by many factors, such as muscular or polyneuropathic changes due to hospitalization, which can affect differently thickness and strength [35, 36].

The HHD overcomes many of the limitations associated with the use of isokinetic dynamometers (considering the gold-standard for the assessment of strength). In fact, while the latter require patients to move to a different location to be tested, well-trained users and expensive initial investment, HHD is portable, fast to use (with measurements done in less than two minutes during consultation/medical evaluation) and displays lower costs. This may propel the measurement of muscle strength in clinical practice which has been very limited so far. However, this requires not only that HHD are demonstrated to be reliable tools, but also that they are useful to estimate patients’ functional level and evolution over time (which requires prospective studies to confirm this theory).

In conclusion, knee extension strength measured with HHD seems to be a reliable resource to objectively measure strength on patients in rehabilitation programs. This method seems feasible to apply, even for physicians with no previous contact with the HHD. Some questions are still to answer, including if method is adequate to objectively measure patients’ functional evolution over time.

## Supporting information

S1 Table Individual intra-rater intra-class correlation coefficients (ICC) of knee extension strength measured with hand-held dynamometers using average, maximum and first values within AB and CD assessments for all the observers. (DOCX)
