# Supplementary material for: Handheld dynamometer reliability to measure knee extension strength in rehabilitation patients—A cross-sectional study
Source: PLoS One. 2022 May 17;17(5):e0268254. doi: 10.1371/journal.pone.0268254 (PMC9113580; doi:10.1371/journal.pone.0268254)
Supplement: S1 Table — (DOCX) [file pone.0268254.s001.docx]

**S1 table: Individual intra-rater intra-class correlation coefficients (ICC) of knee extension strength measured with hand-held dynamometers using Average, Maximum and First values within AB and CD assessments for all the observers**

|  | ICC calculated based on average of measurements within each assessment (95%CI) | ICC calculated based on maximum of measurements within each assessment (95%CI) | ICC calculated based on first measurements within each assessment (95%CI) |
| --- | --- | --- | --- |
| Knee Extension Strength intra-rater ICC |  |  |  |
| Experienced Observer 1 | 0.980 (0.953-0.991) | 0.952 (0.895-0.978) | 0.949 (0.887-0.977) |
| Experienced Observer 2 | 0.982 (0.963-0.992) | 0.972 (0.943-0.987) | 0.950 (0.897-0.976) |
| Inexperienced Observer 1 | 0.973 (0.943-0.987) | 0.957 (0.911-0.980) | 0.955 (0.906-0.978) |
| Inexperienced Observer 2 | 0.980 (0.957-0.991) | 0.965 (0.927-0.983) | 0.948 (0.892-0.975) |

95%CI – 95% Confidence Interval
